# A Milk Test

**Published:** 1879-12

**Authors:** 


A Milk Test.—A German paper gives a test for watered milk, which is simplicity itself. A well-polished knitting-needle is dipped into a deep vessel of milk, and immediately withdrawn in an upright position. If the sample is pure, some of the fluid will hang to the needle; but if water has been added to the milk, even in small proportions, the fluid will not adhere to the needle.—*Cin. Med. News.*

